# Associations of social and material deprivation with tobacco, alcohol, and psychotropic drug use, and gender: a population-based study

**DOI:** 10.1186/1476-072X-6-50

**Published:** 2007-11-09

**Authors:** Michèle Baumann, Elisabeth Spitz, Francis Guillemin, Jean-François Ravaud, Marie Choquet, Bruno Falissard, Nearkasen Chau, Lorhandicap group

**Affiliations:** 1 INtegrative research unit on Social and Individual DEvelopment (INSIDE), University of Luxembourg, Faculty LSHASE, Luxembourg; Luxembourg;; 2 Department of psychology, University of Metz, Metz, France;; 3 EA 4003, Ecole de Santé Publique, University Henri Poincaré – Nancy 1, Faculté de Médecine, Vandoeuvre-lès-Nancy, France;; 4 INSERM, U 750, CERMES, IFR25-IFRH, Villejuif, France;; 5 INSERM, U669, Paris, France;; 6 Univ Paris-Sud, Paris, France;; 7 Univ Paris-Descartes, UMR-S0669 Paris, France;; 8 AP-HP, Villejuif, France.

## Abstract

**Background:**

The aim was to assess the relationships between social and material deprivation and the use of tobacco, excessive alcohol and psychotropic drugs by both sexes and in various age groups. Greater knowledge concerning these issues may help public health policy-makers design more effective means of preventing substance abuse.

**Methods:**

The sample comprised 6,216 people aged ≥ 15 years randomly selected from the population in north-eastern France. Subjects completed a post-mailed questionnaire covering socio-demographic characteristics, occupation, employment, income, smoking habit, alcohol abuse and "psychotropic" drug intake (for headache, tiredness, nervousness, anxiety, insomnia). A deprivation score (D) was defined by the cumulative number of: low educational level, manual worker, unemployed, living alone, nationality other than western European, low income, and non-home-ownership. Data were analysed using adjusted odds ratios (ORa) computed with logistic models.

**Results:**

Deprivation was common: 37.4% of respondents fell into category D = 1, 21.2% into D = 2, and 10.0% into D ≥ 3. More men than women reported tobacco use (30.2% vs. 21.9%) and alcohol abuse (12.5% vs. 3.3%), whereas psychotropic drug use was more common among women (23.8% vs. 41.0%). Increasing levels of deprivation were associated with a greater likelihood of tobacco use (ORa vs. D = 0: 1.16 in D = 1, 1.49 in D = 2, and 1.93 in D ≥ 3), alcohol abuse (1.19 in D = 1, 1.32 in D = 2, and 1.80 in D ≥ 3) and frequent psychotropic drug intake (1.26 in D = 1, 1.51 in D = 2, and 1.91 in D ≥ 3). These patterns were observed in working/other non-retired men and women (except for alcohol abuse in women). Among retired people, deprivation was associated with tobacco and psychotropic drug use only in men.

**Conclusion:**

Preventive measures should be designed to improve work conditions, reduce deprivation, and help deprived populations to be more aware of risk and to find remedial measures.

## Background

Worldwide, the use of tobacco, alcohol and psychotropic drugs results in substantial morbidity and mortality [1-6]. More than 400,000 people die from cigarette smoking every year, and one in every five deaths in the United States is believed to be smoking-related [7]. The consequences of smoking include respiratory and cardiovascular diseases, cancer, physical disabilities, mental disorders, injury, and death [1,8-14]. Among the effects of alcohol abuse are cirrhosis, cardiovascular disease, cancer, gastrointestinal problems, neurocognitive deficits, bone loss, emotional challenges, depression, deterioration in posture control and mobility, injury, job-loss and premature death [4,15-17]. Psychotropic drug intake is common in Europe [1,2,5,18] and alters health status, increases the risk of cancer, injury, and obesity, and deteriorates quality of life [3,13,19-22]. Substance abuse is associated with poor living conditions [2], and recent research has shown that its aetiology involves genetic, material, social and psychological factors [2]. Use of tobacco, alcohol or psychotropic drugs is widely recognised to be a strong, but controllable, risk factor for poor health and social disparities in health [1,3-5,8,23]. It is therefore necessary, from a pubic health perspective, to identify and help those individuals most at risk.

Social and material deprivation affects a large number of people [24-27] and is multidimensional. Factors to consider include low educational level, poor employment status (manual worker, unemployed), living alone, ethnic background (other than western European) low income, and not being a home-owner [28-33]. Findings over recent decades have shown that, taken together, these characteristics result in very difficult living conditions, markedly deteriorated health status, premature death, and unsafe health-related behaviours [8,24-40], notably substance use [1,2,8,23,25,35,36].

Most studies in the literature were focused on one substance, but the patterns of risk may vary from substance to substance due to differences in their social acceptability and other factors. Greater understanding in this area would be expected to lead to improvements in the design of preventive measures. France is a vine growing country where alcohol consumption and psychotropic drug intake are higher than elsewhere, and smoking is prevalent[1,2,5].

Risk patterns may also differ between working and retired people and between the sexes. Many working people, particularly manual workers, use substances in order to cope with work-related difficulties [23,41], and occupations differ between men and women. On the whole, retired people have less good health status and more disability[26,42] than working people due to ageing itself and to having a longer history of working. Risk patterns also vary according to occupational group. Women suffer more from mental disorders than do men[25,42,43], and are more likely to take psychotropic drugs under medical supervision [1,17], to be given them following a medical consultation, to receive longer courses, and to renew the treatment [44].

Key questions in this context concern the relationships between deprivation and tobacco, alcohol and psychotropic drug use. Sex- and age-related differences among active and retired people are also of interest, as are the associations between various dimensions of deprivation and the use of specific substances. Knowledge about these patterns may be of use to professionals directly helping the people concerned, and to those responsible for designing and implementing public health policies intended to reduce social inequalities. Disparities in health are a burgeoning field in which debate is ongoing concerning the models used to explain inequality (particularly whether it is mediated by social, economic and lifestyle-related determinants of health) [29,32,45]. Although social disparities in mortality are greater in France than in other western European countries [34], inequalities in health are poorly documented there [46-48]. Research in recent decades has shed light on the impact of deprivation on health, but most of the work has focused on populations in specific geographic areas defined by neighbourhood deprivation indices [28-30]. Deprivation affects people in most parts of all countries, and epidemiological studies may need to look beyond the so-called "deprived areas" [24,25].

The present study looked at the relationship between social deprivation and substance use among people aged 15+ years in a French population. It investigated (1) the relationships between deprivation and tobacco, excessive alcohol and psychotropic drug use, (2) sex and age differences among working/other non-retired and retired people, and (3) the relationships between various aspects of deprivation, and tobacco, excessive alcohol and psychotropic drug use.

## Materials and methods

The initial sample consisted of everyone aged 15 years or more living in 8,000 randomly selected households in the Lorraine region of north-eastern France (2.3 million inhabitants). Only households with a telephone were eligible.

Before the initial survey, a 3-month media campaign (television, print, and radio) was conducted in order to raise awareness. The investigation was approved by the *Commission Nationale d'Informatique et Libertés*, and written informed consent was obtained from respondents.

The study protocol included: (a) an application to participate that ascertained the number of people in the household, and (b) three standardized self-administered questionnaires with a covering letter and a pre-paid envelope for the reply. Mailings were made at 1-month intervals. When the number of individuals was unknown, two questionnaires were sent first, and another later. Questionnaires were completed by the subjects themselves, but adolescents were free to ask their parents about anything they did not understand. Questions covered: sex, date of birth, educational level, occupation (previous occupation for retired people) coded according to the Insee classification (Paris, 1983), smoking habit, alcohol abuse, nationality, family characteristics, unemployment, home-ownership, perceived income, and psychotropic drug use. Alcohol abuse was defined using the Deta questionnaire (at least two positive responses to four items: (i) consumption considered excessive by the subject; (ii) consumption considered excessive by people around the subject, (iii) subject wishes to reduce consumption, and (iv) consumption on waking) [8,25]. With regard to perceived income, subjects were asked whether they considered themselves: comfortable or well off, earning just enough, coping but with difficulties, or getting into debt. Psychotropic drug use was determined by asking whether respondents had frequently taken medication (prescribed and/or non-prescribed) for headache, tiredness, nervousness or anxiety, or insomnia over the previous year (Yes/No) [8,18,43]. Deprivation was defined by the number of positive responses to the following seven criteria: low educational level (primary school), manual worker, unemployed, living alone, nationality other than western European, low income (coping, but with difficulties, or getting into debt), and not being a home-owner [28,30].

Of the 8,000 households included in the sample, mailings to 193 (2%) were lost (due to address error or death). Of 7,807 households contacted, 3,460 (44.3%) participated (all eligible members of the family took part in 86% of those). In total, 6,234 subjects filled in a questionnaire; 18 were of unknown sex or age, leaving 6,216 subjects who were similar in age and sex distribution to the Lorraine population (Table 1).

**Table 1:** Distribution according to sex and age of the sample studied and of the general population of Lorraine [49] (%)

	Sample studied	Lorraine general population
No. of subjects	6,216	1,848,579
Percentage of women	52.4	51.5
Age (yr)		

15–19	5.4	9.6
20–24	8.0	9.8
25–29	9.7	9.7
30–34	10.4	9.6
35–39	10.5	9.6
40–44	7.9	9.3
45–49	8.5	5.9
50–54	6.0	6.6
55–59	6.3	6.8
60–64	7.2	6.6
65–69	7.5	5.7
70 or over	12.6	10.8

Only people aged 15 or more were considered.

### Statistical analyses

The χ^2 ^independence test was used to compare the two sexes for various variables. The deprivation score (D) was defined by the number of the seven criteria considered above and classified into four groups: D = 0, D = 1, D = 2, and D ≥ 3. We also used the principal component analysis in order to define a score as a linear combination of the previous criteria but this was not retained because: (1) the seven eigenvalues found were close enough (1.33, 1.20, 1.06, 0.97, 0.87, 0.84, and 0.73), suggesting that each component contributed almost equally to the D; and (2) the relationships between the deprivation levels defined by the 50^th^, 75^th^, and 90^th ^percentile values of the score obtained [49] with tobacco, excessive alcohol, and psychotropic drug use were close to those found with D = 0, D = 1, D = 2, and D ≥ 3. Cronbach's alpha was modest (0.24). To assess the relationships between D and tobacco, excessive alcohol and psychotropic drug use, adjusted odds ratios (ORa) and 95% confidence intervals were calculated using logistic models.

## Results

The characteristics of the subjects are shown in Table 2. Deprivation (D) was common: 37.4% of subjects were classified as D = 1, 21.2% D = 2, and 10.0% D ≥ 3. Low educational level, living alone, unemployment, and not being a home-owner were more common among women, whereas men were more likely to be manual workers. Men exhibited a significantly (p < 0.001) higher prevalence than women of tobacco use (30.2% vs. 21.9%) and alcohol abuse (12.5% vs. 3.3%), but women reported more frequent psychotropic drug use (for headache, tiredness, nervousness/anxiety, insomnia, 41.0% vs. 23.8%).

**Table 2:** Characteristics of the subjects by sex: %

	*Men*	*Women*	*p-value*
	*(n = 2,959)*	*(n = 3,257)*	
Age (yr)			*0.002*
*≤29*	*21.5*	*24.5*	
*30–39*	*21.8*	*20.1*	
*40–49*	*17.8*	*15.1*	
*50–59*	*12.6*	*12.0*	
*60–69*	*14.5*	*15.0*	
*≥70*	*11.8*	*13.3*	
			
Deprivation (D)			
*Low educational level (primary school)*	*26.4*	*32.1*	*<0.001*
*Manual worker*	*27.0*	*9.5*	*<0.001*
*Low perceived income (with difficulties)*	*8.8*	*9.0*	*0.74*
*Living alone*	*7.7*	*15.0*	*<0.001*
*Unemployed*	*3.1*	*4.6*	*<0.001*
*Foreign nationality (other than Western European)*	*2.4*	*1.8*	*0.11*
*Not home-owner*	*36.9*	*40.9*	*<0.001*
			
*D score*			*0.01*
*0*	*32.9*	*29.9*	
*1*	*35.7*	*39.1*	
*2*	*20.9*	*21.5*	
*≥3*	*10.5*	*9.5*	
			
*Tobacco use*	*30.2*	*21.9*	*<0.001*
*Alcohol abuse*	*12.5*	*3.3*	*<0.001*
			
Frequent use of psychotropic drugs	23.8	41.0	*<0.001*
*Tiredness*	*3.1*	*6.1*	*<0.001*
*Headache*	*13.7*	*25.9*	*<0.001*
*Insomnia*	*6.2*	*11.0*	*<0.001*
*Nervousness or anxiety*	*5.9*	*13.6*	*<0.001*

Table 3 shows that the deprivation patterns differed between various age groups with the exception of "foreign" nationality for both sexes. There were clear differences between generations/ages in educational level, income and home-ownership. Tobacco use was more frequent among younger men and women. Alcohol abuse predominantly affected men aged 40–59 and women aged under 50. No difference between the age groups was found in psychotropic drug use among either men or women.

**Table 3:** Relationship between age and deprivation (D) and substance use for each sex: %

	*Men (2,959 subjects)*							*Women (3,257 subjects)*						
		
	*≤29*	*30–39*	*40–49*	*50–59*	*60–69*	*≥70*	*p-value*	*≤29*	*30–39*	*40–49*	*50–59*	*60–69*	*≥70*	*p-value*
*No. of subjects*	*637*	*645*	*528*	*373*	*428*	*348*		*799*	*655*	*491*	*391*	*487*	*434*	
*Deprivation*														
*Low educational level*	*10.1*	*15.2*	*22.7*	*29.0*	*44.2*	*58.0*	*<0.001*	*8.8*	*16.5*	*30.5*	*44.5*	*56.5*	*61.7*	*<0.001*
*Manual worker*	*20.4*	*35.6*	*29.4*	*28.2*	*25.2*	*20.1*	*<0.001*	*6.4*	*9.9*	*12.4*	*9.0*	*12.3*	*8.8*	*0.002*
*Low perceived income*	*11.9*	*9.6*	*11.2*	*9.9*	*3.7*	*2.6*	*<0.001*	*10.9*	*9.3*	*13.9*	*8.4*	*4.3*	*5.3*	*<0.001*
*Living alone*	*10.1*	*7.9*	*5.9*	*5.1*	*6.5*	*10.1*	*0.012*	*9.4*	*7.0*	*4.1*	*13.0*	*22.6*	*42.6*	*<0.001*
*Unemployed*	*5.0*	*3.4*	*2.5*	*5.9*	*0.2*	*-*	*<0.001*	*7.3*	*5.5*	*5.3*	*5.9*	*1.4*	*-*	*<0.001*
*Foreign nationality*	*2.4*	*2.0*	*2.1*	*2.7*	*2.6*	*3.4*	*0.79*	*1.8*	*1.7*	*0.8*	*3.1*	*1.9*	*2.3*	*0.24*
*Not home-owner*	*56.4*	*48.7*	*31.2*	*22.8*	*18.0*	*26.2*	*<0.001*	*61.2*	*46.7*	*31.0*	*28.4*	*24.2*	*36.2*	*<0.001*
D score							*<0.001*							*<0.001*
*D = 1*	*39.3*	*37.0*	*31.2*	*32.7*	*31.3*	*42.0*		*42.5*	*38.9*	*31.4*	*37.6*	*42.7*	*38.7*	
*D = 2*	*23.7*	*22.0*	*18.0*	*15.3*	*21.0*	*23.8*		*21.3*	*16.3*	*18.5*	*18.9*	*24.8*	*32.0*	
*D ≥ 3*	*9.1*	*12.6*	*11.4*	*11.3*	*8.6*	*9.8*		*6.4*	*7.9*	*9.0*	*11.3*	*9.9*	*16.4*	
*Tobacco use*	*37.1*	*41.2*	*38.1*	*23.6*	*15.0*	*11.2*	*<0.001*	*34.7*	*36.0*	*21.4*	*12.0*	*7.2*	*3.0*	*<0.001*
*Alcohol abuse*	*11.0*	*11.8*	*15.9*	*16.6*	*10.8*	*8.9*	*0.003*	*3.9*	*4.0*	*5.1*	*3.1*	*1.4*	*1.4*	*0.004*
*Frequent use of psychotropic drugs*	*21.5*	*23.7*	*25.2*	*23.3*	*22.0*	*28.4*	*0.19*	*42.2*	*36.0*	*41.3*	*43.2*	*41.1*	*43.5*	*0.10*

Age was related to D with p < 0.001.

The results in Tables 4 and 5 demonstrate a strong and similar relationship between deprivation and tobacco and psychotropic drug use among both sexes, and with alcohol abuse in men (not women) of all ages combined. However, risk patterns differed between working/other non-retired subjects and retired respondents. Among working/other non-retired men and women, the relationship between deprivation and tobacco and psychotropic drug use persisted, whatever the age group. The risk of alcohol abuse was similar for the D = 1, D = 2 and D ≥ 3 groups in men and non-significant in women. In retired men, there was a proportional relationship between deprivation and psychotropic drug use, but only the D ≥ 3 group had a significant OR for tobacco and alcohol abuse. Among retired women, a higher risk of psychotropic drug use was observed in subjects who were in D ≥ 3 and less than 70 years old.

**Table 4:** Relationships between deprivation score (D)and tobacco, excessive alcohol, and frequent psychotropic drug use for various age groups among working and other non-retired people: odds ratios and 95% CI, vs. D = 0

	*Working and other non-retired people *^*a*^						*All the sample *^*b*^	
			
	*Age < 40 yr*		*Age ≥ 40 yr*		*Total*			
***Men***								
No. of subjects	986		845		1831		2959	
**Tobacco use**								
D = 1	1.47*	1.04–2.07	1.42*	1.00–2.03	1.52‡	1.20–1.94	1.27*	1.04–1.56
D = 2	1.98‡	1.36–2.88	1.75†	1.16–2.65	2.00‡	1.53–2.63	1.73‡	1.37–2.18
D ≥ 3	3.43‡	2.20–5.37	1.98†	1.23–3.19	2.83‡	2.05–3.88	2.62‡	1.98–3.46
**Excessive alcohol use**								
D = 1	2.09†	1.18–3.71	1.62*	1.02–2.57	1.68‡	1.19–2.39	1.46†	1.10–1.94
D = 2	1.88*	1.01–3.52	1.83*	1.07–3.10	1.66†	1.12–2.46	1.54†	1.12–2.12
D ≥ 3	1.97	0.97–4.01	2.29†	1.28–4.09	1.95†	1.25–3.03	2.26‡	1.58–3.23
**Psychotropic drug use**								
D = 1	1.03	0.68–1.54	1.55*	1.05–2.28	1.24	0.94–1.64	1.32*	1.06–1.65
D = 2	1.64*	1.07–2.50	2.35‡	1.52–3.64	1.91‡	1.42–2.58	1.71‡	1.34–2.17
D ≥ 3	2.18†	1.34–3.53	2.40‡	1.46–3.96	2.30‡	1.63–3.24	2.22‡	1.67–2.96
***Women***								
No. of subjects	1096		1094		2190		3257	
**Tobacco use**								
D = 1	1.25	0.93–1.70	0.97	0.64–1.47	1.18	0.93–1.49	1.21	0.98–1.51
D = 2	2.12‡	1.49–3.00	0.99	0.61–1.61	1.60‡	1.22–2.09	1.75‡	1.36–2.26
D ≥ 3	2.14‡	1.35–3.39	2.06†	1.20–3.51	2.01‡	1.43–2.82	2.06‡	1.48–2.86
**Excessive alcohol use**								
D = 1	0.91	0.40–2.06	1.40	0.66–2.91	1.15	0.66–1.98	1.11	0.68–1.79
D = 2	1.52	0.65–3.56	0.91	0.35–2.36	1.21	0.64–2.26	1.43	0.84–2.44
D ≥ 3	0.62	0.14–2.86	0.84	0.23–3.04	0.74	0.28–1.97	1.10	0.51–2.33
**Psychotropic drug use**								
D = 1	1.04	0.77–1.40	1.13	0.85–1.52	1.08	0.88–1.33	1.16	0.97–1.38
D = 2	1.20	0.85–1.69	1.53*	1.09–2.14	1.35*	1.06–1.72	1.36†	1.11–1.66
D ≥ 3	1.44	0.91–2.28	2.23‡	1.43–3.46	1.81‡	1.32–2.48	1.77‡	1.36–2.30

*P < 0.05, †P < 0.01, ‡P < 0.001.

^*a *^Students were excluded (n = 643). ^*b *^Odds ratios adjusted for age.

**Table 5:** Relationships between deprivation score (D)and tobacco, excessive alcohol, and frequent psychotropic drug use for various age groups among retired people: odds ratios and 95% CI, vs. D = 0

	*Retired people*						*All the sample *^*a*^	
			
	*Age < 70 yr*		*Age ≥ 70 yr*		*Total*			
***Men***								
No. of subjects	503		333		836		2959	
**Tobacco use**								
D = 1	1.04	0.59–1.82	0.63	0.23–1.69	0.81	0.50–1.32	1.27*	1.04–1.56
D = 2	1.23	0.65–2.30	1.97	0.77–4.99	1.36	0.82–2.26	1.73‡	1.37–2.18
D ≥ 3	2.00	0.95–4.21	2.03	0.64–6.38	1.88*	1.01–3.48	2.62‡	1.98–3.46
**Excessive alcohol use**								
D = 1	0.94	0.47–1.88	0.86	0.29–2.51	0.86	0.48–1.52	1.46†	1.10–1.94
D = 2	1.41	0.68–2.92	1.03	0.32–3.33	1.20	0.65–2.22	1.54†	1.12–2.12
D ≥ 3	3.31†	1.50–7.28	2.78	0.82–9.35	2.97‡	1.54–5.72	2.26‡	1.58–3.23
**Psychotropic drug use**								
D = 1	1.53	0.90–2.61	1.40	0.74–2.65	1.56*	1.05–2.34	1.32*	1.06–1.65
D = 2	1.65	0.91–3.00	1.35	0.66–2.77	1.59*	1.01–2.51	1.71‡	1.34–2.17
D ≥ 3	2.00	0.95–4.21	2.28	0.95–5.45	2.21†	1.26–3.87	2.22‡	1.67–2.96
***Women***								
No. of subjects	368		285		716		3257	
**Tobacco use**								
D = 1	1.75	0.60–5.09	(1)		1.03	0.43–2.46	1.21	0.98–1.51
D = 2	2.77	0.94–8.23	0.34	0.07–1.77	1.34	0.55–3.25	1.75‡	1.36–2.26
D ≥ 3	0.94	0.17–5.04	0.40	0.08–2.05	0.33	0.07–1.57	2.06‡	1.48–2.86
**Excessive alcohol use**								
D = 1	0.65	0.04–10.5	(1)		0.17	0.02–1.63	1.11	0.68–1.79
D = 2	2.14	0.19–24.0	0.40	0.06–2.96	0.93	0.21–4.23	1.43	0.84–2.44
D ≥ 3	2.38	0.15–39.1	0.72	0.10–5.32	1.37	0.27–6.93	1.10	0.51–2.33
**Psychotropic drug use**								
D = 1	1.29	0.75–2.22	1.04	0.52–2.05	1.21	0.80–1.85	1.16	0.97–1.38
D = 2	1.39	0.76–2.51	1.04	0.52–2.10	1.26	0.81–1.97	1.36†	1.11–1.66
D ≥ 3	2.91†	1.36–6.23	1.00	0.46–2.16	1.65	0.98–2.76	1.77‡	1.36–2.30

^a^Odds ratio adjusted for age.

Tables 6 and 7 show that risk patterns varied between working/other non-retired and retired people, the two sexes, and according to the deprivation dimensions and the substance concerned. Among working/other non-retired men, low educational level, manual employment and low income were related to tobacco and psychotropic drug use, whereas low income was associated with the use of all three substances, living alone with alcohol abuse only; and not being a home-owner with alcohol abuse. Among retired men, having been a manual worker was associated with alcohol abuse only, and low income with alcohol abuse and psychotropic drug use. In women, low educational level, being a manual worker and low income were associated with psychotropic drug use, but low income, living alone and unemployment were associated with tobacco use in working/other non-retired and retired subjects. Among retired women a significant association was noted between not being a home-owner and alcohol abuse only.

**Table 6:** Relationships between deprivation score (D)and tobacco, excessive alcohol, and frequent psychotropic drug use among working and other non-retired people: odds ratios adjusted for age and 95% CI

	*Working and other non-retired people*					
	
	*Tobacco use*		*Excessive alcohol intake*		*Frequent use of psychotropic drugs*	
***Men (1831 subjects)***						
Deprivation (D)						
Low educational level	1.39†	1.08–1.79	1.02	0.72–1.45	2.05‡	1.58–2.66
Manual worker	1.75‡	1.42–2.14	1.21	0.91–1.61	1.49‡	1.19–1.86
Low perceived income	1.69‡	1.25–2.28	2.20‡	1.54–3.16	1.96‡	1.44–2.67
Living alone	1.25	0.87–1.79	1.78†	1.14–2.76	1.39	0.95–2.04
Unemployed	1.43	0.92–2.22	1.31	0.73–2.34	1.00	0.61–1.64
Foreign nationality	1.13	0.58–2.21	0.53	0.16–1.77	1.76	0.91–3.41
Not home-owner	1.50‡	1.23–1.83	1.46†	1.10–1.93	1.11	0.89–1.38
***Women (2190 subjects)***						
Deprivation						
Low educational level	1.08	0.84–1.39	0.95	0.55–1.64	1.42†	1.15–1.74
Manual worker	1.13	0.82–1.56	0.69	0.30–1.62	1.34*	1.01–1.77
Low perceived income	2.50‡	1.86–3.36	1.07	0.52–2.18	1.56†	1.18–2.06
Living alone	1.68†	1.20–2.36	1.74	0.87–3.48	1.10	0.82–1.47
Unemployed	1.53*	1.07–2.20	0.95	0.37–2.39	0.92	0.65–1.30
Foreign nationality	0.45	0.17–1.19	(1)		1.36	0.72–2.58
Not home-owner	center1.43‡	1.17–1.76	1.04	0.64–1.68	1.14	center0.95–1.37
*P<0.05, †P<0.01, ‡P<0.001						

**Table 7:** Relationships between deprivation score (D)and tobacco, excessive alcohol, and frequent psychotropic drug use among retired people: odds ratios adjusted for age and 95% CI

	*Retired people*					
	*Tobacco use*		*Excessive alcohol intake*		*Frequent use of psychotropic drugs*	
***Men (836 subjects)***						
Deprivation (D)						
Low educational level	1.37	0.93–2.03	1.23	0.79–1.94	1.29	0.93–1.78
Manual worker	1.27	0.83–1.96	2.22‡	1.40–3.52	1.32	0.92–1.90
Low perceived income	1.80	0.82–3.95	2.45*	1.08–5.56	2.20*	1.07–4.54
Living alone	1.58	0.81–3.10	1.65	0.77–3.52	1.13	0.63–2.04
Unemployed	-		-		-	
Foreign nationality	1.27	0.42–3.84	(1)		1.11	0.43–2.87
Not home-owner	1.38	0.88–2.16	1.57	0.95–2.60	1.38	0.95–2.01
***Women*** (716 subjects)						
Deprivation						
Low educational level	0.70	0.36–1.35	0.46	0.24–1.65	1.09	0.80–1.48
Manual worker	0.70	0.24–2.04	1.77	0.37–8.45	1.48	0.95–2.32
Low perceived income	0.89	0.20–4.03	(1)		1.13	0.56–2.27
Living alone	1.23	0.61–2.47	1.66	0.48–5.76	1.23	0.90–1.70
Unemployed	-		-		-	
Foreign nationality		3.45	center0.69–17.2	(1)	0.55	0.17–1.77
Not home-owner	1.43	0.72-2.87	4.46*	1.27-15.7	1.09	0.78-1.52
*P<0.05, ‡P<0.001					

## Discussion

The present study demonstrates that deprivation is common in the population considered, and that it has a strong association with tobacco, excessive alcohol intake, and psychotropic drug use. It highlights that risk patterns vary with the substance concerned, sex, and between active and retired people. The risk of substance use differed between deprivation dimensions. The material and social deprivation index used here was defined from seven criteria (low educational level, manual worker, unemployed, living alone, nationality other than western European, low income, and not being a home-owner) generally used in the literature [28,30]. The nationality criterion was included in the deprivation index considered because it may be associated with cultural disadvantages, poor work/living conditions, poor living environment, poor health and access to care. It should be noted that racial composition has been included in deprivation indexes by several authors [30]. The seven eigenvalues found with the principal component analysis were close enough (between 0.73 and 1.33) to suggest that all components contributed almost equally to the D value. Cronbach's alpha was modest. A similar observation was highlighted in the literature after elimination of redundant items in each domain[30]. The deprivation index described here reflects the multidimensional character of community socioeconomic status [30].

These findings indicate that material and social conditions are potential risk factors for harmful health-related behaviours during both working life and retirement, and that the presence of several dimensions of deprivation is associated with a very high risk. This is consistent with the results of other studies, although, to our knowledge, they did not focus on all three substances studied here [19,23,28-30,32]. Tobacco, alcohol and psychotropic drugs are strong contributors to social disparities in health [1,3-5,8,23]. Two studies in France showed a strong relationship of the cumulative number of deprivation dimensions with tobacco, cannabis, psychotropic, tranquillizer and antidepressant use, as well as with physical and mental health status, obesity, underweight, diabetes and hypertension [5,24].

Any selection bias here would be small: 96% of households had telephones at the time of the study, and only 16% had confidential addresses. Discussions before the survey, for example with associations of people with disabilities, suggested that this list would not be biased with regard to health status or living conditions. The participation rate was rather modest but similar to that achieved in similar surveys in France [1,49]. The age and sex distributions of the sample reflect those of the general population of Lorraine [50]. The quality of the completed questionnaires was very good. As mentioned above, all the factors studied had been validated and used in other studies [5,8,13,18,43].

Although the study was conducted on a large sample, the results should be interpreted with caution due to possible selection bias. The self-administered occupational health history questionnaire is considered reliable and valid [51]. A study analysing non-response bias in a mailed health survey showed that respondents and non-respondents were of similar sex and age distributions, and close in terms of health care expenditure [52]. Similar observations were reported by the Maastricht Cohort Study [53]. The prevalences of various variables in the present sample were similar to the directly standardized adjusted rates computed in reference to the Lorraine population [50]. This is due, as noted above, to similar age and sex distributions in the sample and the Lorraine population as a whole. It should be noted that our study would underestimate the differences, as the most economically deprived (i.e. those with no home and therefore no telephone) were not included in the sample.

Our study found that 30% of men and 22% of women were current smokers. These figures differ slightly from those reported among French people aged 12–75 in 1999 (32% and 26% respectively) [25]. Alcohol abuse (in terms of the Deta index) was similar at 13.3% in men and 4% in women [25]. The higher prevalence of alcohol abuse among men seen here was also found in the ESEMeD study [42]. Psychotropic drug use was common (23.8% in men and 41.0% in women). Comparison with other studies is difficult because of variations in the study populations, the psychotropic drugs considered, and the methodological approaches adopted. In France, one-third of workers use drugs for work-related reasons, 20% to feel better, 12% to control an awkward symptom, and 18% to relax after a difficult day's work [41,54]. The ESEMeD study found that the prevalence of antidepressant, anxiolytic, and antipsychotic or mood-stabilizing drug use over a 12-month period was 19.2% in France, 15.5% in Spain, 13.7% in Italy, 13.2% in Belgium, 7.4% in the Netherlands, and 5.9% in Germany [1]. In Belgium, Bruffaerts et al. [55] reported that about 19% of people aged 18 years or more said they had used a psychotropic drug over the previous 12 months.

The present study reveals a strong association between deprivation and tobacco and psychotropic drug use, for the two age groups (<40 and ≥40 years), among working/other non-retired men and women. In other words, the likelihood of tobacco and psychotropic drug use increases with the deprivation score, and the association appears early and persists throughout the working lives of both sexes. These findings are consistent with those of other studies. It is reported that one-third of the French working population use medications or other legal psychoactive substances in order to cope with work-related difficulties, and that such use is more common in manual workers [41]. Manual workers have poorer working conditions that may lead to physical and mental disturbances [26] and consequently to psychotropic drug use. Manual workers also have a higher prevalence of tobacco use [2,5] and of disabilities than other workers in both age groups <40 and ≥40 years [26]. Physical job demands lead to fatigue [56] and the development of work-related stress reactions, psychological overload, and health problems [57]. Cumulative job stress is common and is associated with increased risk of mental health disorders and psychotropic drug use [58]. The volume of services provided and job dissatisfaction are associated with hypnotic and tranquillizer use [59]. Interesting findings here were that among the seven deprivation domains studied: (1) low educational level, being a manual worker and low income were associated with psychotropic drug use among men as well as women; (2) tobacco use was related to low educational level, being a manual worker, low income and not being a home-owner in men, and to low income, living alone, unemployment and not being a home-owner in women. Other studies have stated that unemployment, low educational level, being a manual worker, being divorced or widowed, and living alone were associated with an increased risk of tobacco and psychotropic drug use in France and in Europe [1,2,5,35,36]. No relationship between nationality and any substance use was observed in our study – this was also true for psychotropic drug use in Europe (when controlling for co-factors) [1].

With regard to alcohol abuse, our study found a strong relationship with deprivation in male, but not female, working/other non-retired people. Alcohol abuse was more common in men than in women (12.5% vs. 3.3%, p < 0.001). This sex difference has also been reported by other studies in France [25,42] which related increasing deprivation to alcohol abuse, but not daily alcohol use [5]. It should be noted that of the seven deprivation criteria considered, only low income, living alone and not being a home-owner were related to alcohol abuse in men, and no association was observed among women. The differences between the sexes may be explained in part by the fact that alcohol abuse is three-fold lower in women, who are more likely to take psychotropic drugs [1,17]. Excessive alcohol use appeared here to be more associated with poverty and poor living conditions [2] than with working issues. Other investigations have found a relationship between living alone and unemployment, and partner-relationship disruptions are strongly associated with suicidal behaviour among individuals with alcoholism [35,36].

Another important finding is that risk patterns for substance use clearly differ between active and retired men, and between retired men and retired women. Indeed, among the active and retired men, there was a gradient in the relationship between psychotropic drug useand deprivation score, whereas smoking and alcohol abuse were associated only with D ≥ 3. The lower overall associations between deprivation and smoking and alcohol abuse among retired men (except D ≥ 3 group for alcohol abuse) may be explained as follows: (1) smoking and alcohol abuse are less frequent in retired people (compared with other generations/age groups); (2) premature mortality (before 65/70 years) is higher among subjects most at risk, particularly manual workers, smokers and alcohol users [37,38] (this was also observed in the 9-year prospective premature mortality (<70 years) analysis of the sample studied: manual workers had an increased adjusted risk ratio of 1.84, 95% CI 1.00–3.37 compared with upper class; risk ratios for current smokers and ex-smokers were 1.76, 95% CI 1.08–2.88 and 1.52, 95% CI 0.96–2.40 respectively; and subjects with alcohol abuse had a risk ratio of 2.07, 95% CI 1.31–3.26 – as yet unpublished data); and (3) the absence of stressful working conditions and a lesser perception of complaints without the physical and mental demands of a job. The strong relationship between psychotropic drug use and deprivation in retired men may be explained by a higher prevalence of altered health status and disability due to aging [26] that could increase psychotropic drug use [1,60,61]. Regarding retired women, only subjects aged less than 70 years with D ≥ 3 had a significant risk for psychotropic drug use (that would be due to manual workers who had a higher consumption, close to significance). It should be noted that only three of the seven deprivation domains were associated with excessive alcohol and psychotropic drug use in retired men: having been a manual worker, low income, and not being a home-owner. Among retired women, only not being a home-owner was related to alcohol abuse. Therefore substance use in retired people relates to previous work conditions and poverty.

The present survey demonstrates that an accumulation of several deprivation dimensions is associated with marked deterioration in health-related behaviours [25,28-32,35,36,39] to cope with very difficult living and working conditions. In many industrialized countries, people start smoking at an increasingly younger age, putting themselves at greater, and earlier, risk of avoidable tobacco-related illnesses [35,62]. Alcohol abuse and psychotropic drug use are very common and often begin at a young age [18,43,62]. Findings over recent decades have shown that multiple deprivations affect many people from childhood onwards, and that the accumulation of various aspects of disadvantages leads to marked deteriorations in living conditions, health-related behaviours and health status [2,8,23,25-32,34-36,39,41]. Observed geographical differences in heath outcomes are attributed to the individual characteristics of members of the populations concerned and their living conditions and lifestyle [63,64]. Some adult health problems and premature mortality may be influenced by the childhood circumstances of the person concerned [2,65,66]. Interventions should be designed and evaluated to address these issues, and the most promising should be implemented on a large scale.

In the present study, women were different from men in that the association between deprivation and substance use was less evident, particularly among retired people. This may be attributable to less job demands, and to differences in the substances consumed in order to deal with everyday life. There were clear differences between age groups in the patterns of both deprivation and substance use. Low educational level was more common among older people, but younger people were more likely to report low income and not being a home-owner. Among men, the proportion of manual workers was higher in the 30–59 age group than in the group aged 60+ years. This tendency can be explained by premature death: for the sample studied between 1996 and 2004, there were 85 deaths in people under 65, and 41 between 65 and 70; premature mortality (<70 years) rates per 1,000 person-years were 1.32, 4.33 and 12.6 for the age groups ≤49, 50–59 and ≥60 years, respectively (data not yet published). Compared with other age groups, men aged 40–69 years were less likely to be living alone, as were women under 50. This phenomenon, which was more marked in women, can in part be explained by the premature death of the spouse. Such changes reflect the deprivation many people face, and the different ways in which it manifests itself over a lifetime.

The results of our study highlight the associations between accumulation of deprivation dimensions and tobacco, alcohol, and psychotropic drug use at various ages. The role of cumulative advantages/disadvantages during the life-time or the mechanism for inequalities across a temporal process are investigated by few authors [67,68]. Ross and Wu [67] showed that among the subjects aged 20 to 64 in the United States, the gap in self-reported health, physical functioning, and physical well-being among people with high and low educational attainment increases with age, and that household income does not explain education's effect. In our study, a stronger relationship in the working and other non-retired people aged 40+ than in those aged 39 or less was found between the deprivation score and psychotropic use only. DiPrete and Eirich [68] examined the different theoretical and empirical cumulative advantages models proposed by sociologists, sociobiologists, social psychologists, and economists as a mechanism for inequality. However, these interesting issues could not be treated in our cross-sectional study. We rather examined the relationships between the accumulation of several deprivation dimensions and tobacco, alcohol, and psychotropic drug use at the time of the survey. Many factors play a role in creating or reducing inequality over time, including: mortality of subjects most at risk, cultural changes, movements of the population (for example north-south for older people, rural migration, etc.), and changes in society as a whole (education levels, living conditions, air pollution, working procedures and hazards, life style, family structure, etc.).

## Conclusion

The present study elucidates the relationships of material and social deprivation with tobacco, excessive alcohol and psychotropic drug use for men and women and in various age-groups. It shows a gradient in the relationship between deprivation and tobacco, excessive alcohol and psychotropic drug use in all age groups during active life and after retirement. Alcohol abuse was related to poverty and family structure, whereas tobacco and psychotropic drug use were related to poverty, educational level and working conditions. Public policies aimed at reducing substance use should address the need to improve physical and mental working conditions, reduce deprivation, and help deprived populations to be more aware of the risks and to find remedial measures.

## Competing interests

The author(s) declare that they have no competing interests.

## Authors' contributions

MB participated in conceiving the study and writing the manuscript.

ES participated in conceiving the study and writing the manuscript.

FG participated in conceiving the study and writing the manuscript.

JFR participated in conceiving the study and writing the manuscript.

MC participated in conceiving the study and writing the manuscript.

BF participated in conceiving the study and writing the manuscript.

NC participated in conceiving the study, carrying out the study and had the main responsibility for writing the manuscript.

Lorhandicap group carried out the study of which this work is a part. The group followed and reviewed the manuscript.

All authors read and approved the final manuscript.
